# Concise Review: Generation of Neurons From Somatic Cells of Healthy Individuals and Neurological Patients Through Induced Pluripotency or Direct Conversion

**DOI:** 10.1002/stem.1782

**Published:** 2014-07-03

**Authors:** Iván Velasco, Patricia Salazar, Alessandra Giorgetti, Verónica Ramos-Mejía, Julio Castaño, Damià Romero-Moya, Pablo Menendez

**Affiliations:** a Instituto de Fisiología Celular-Neurociencias, Universidad Nacional Autónoma de MéxicoMéxico, D.F, México; b Centro GENYOGranada, Spain; c Josep Carreras Leukemia Research Institute, Cell Therapy Program, University of BarcelonaBarcelona, Spain; d Instituciò Catalana de Recerca i Estudis Avançats (ICREA)Barcelona, Spain

**Keywords:** Induced pluripotent stem cells, Neural differentiation, Induced neurons, Direct conversion, Neurodegenerative diseases, Genome editing

## Abstract

Access to healthy or diseased human neural tissue is a daunting task and represents a barrier for advancing our understanding about the cellular, genetic, and molecular mechanisms underlying neurogenesis and neurodegeneration. Reprogramming of somatic cells to pluripotency by transient expression of transcription factors was achieved a few years ago. Induced pluripotent stem cells (iPSC) from both healthy individuals and patients suffering from debilitating, life-threatening neurological diseases have been differentiated into several specific neuronal subtypes. An alternative emerging approach is the direct conversion of somatic cells (i.e., fibroblasts, blood cells, or glial cells) into neuron-like cells. However, to what extent neuronal direct conversion of diseased somatic cells can be achieved remains an open question. Optimization of current expansion and differentiation approaches is highly demanded to increase the differentiation efficiency of specific phenotypes of functional neurons from iPSCs or through somatic cell direct conversion. The realization of the full potential of iPSCs relies on the ability to precisely modify specific genome sequences. Genome editing technologies including zinc finger nucleases, transcription activator-like effector nucleases, and clustered regularly interspaced short palindromic repeat/CAS9 RNA-guided nucleases have progressed very fast over the last years. The combination of genome-editing strategies and patient-specific iPSC biology will offer a unique platform for in vitro generation of diseased and corrected neural derivatives for personalized therapies, disease modeling and drug screening. Stem Cells *2014;32:2811–2817*

## Induced Pluripotency

Pluripotency is defined by the ability to produce from a single undifferentiated cell type, cell derivatives representing the three germ layers: meso-, endo-, and ectoderm. Pluripotent stem cells (PSCs) of human origin were first isolated from teratocarcinomas, giving rise to embryonal carcinoma cell lines 1. This property is expected to be present in cells giving rise to gametes during human development and thus, pluripotent embryonic germ cells were described in 1998 2. The most widely used PSCs of mammalian origin are mouse embryonic stem cells (ESCs), derived from the inner cell mass of preimplantation embryos at the blastocyst stage 3,4. Using a similar approach, human ESCs (hESCs) were isolated from embryos almost 2 decades later 5. During human development, germline cells undergo meiosis to produce gametes. The remaining cellular types are known as somatic cells, and they do not contribute to reproductive tissues. Early work has shown that miss-expression of MyoD can convert cell fate between germ layers 6,7. Similarly, ectopic expression of master lineage-specific transcriptional regulators allows cells to change from one blood lineage to another 8. However, it was not recognized until recently how relatively easy is for a terminally differentiated somatic cell to re-acquire pluripotency properties or change cell fate across germ layers.

Successful reprogramming of somatic cells to a pluripotent state by transient expression of four transcription factors (OCT4, SOX2, KLF4, and c-MYC) was achieved for the first time with mouse cells in 2006 9 and with human fibroblasts in 2007 10,11 using retroviral vectors. Many researchers have produced induced pluripotent stem cells (iPSCs) by expressing the Yamanaka factors through integrative or nonintegrative methods. Among the former, retroviral and lentiviral vectors are the most widely used 12. Induced pluripotency requires endogenous activation of a pluripotency-associated gene expression signature and a concomitant silencing of ectopic reprogramming factors. Genomic integration of these transgenes is random and becomes epigenetically regulated so that their residual expression after long-term culture due to a lack of complete silencing may prevent subsequent differentiation 13 and make iPSCs prone to genomic alterations 14. The most common nonintegrative methodologies include Sendai virus (SeV) 15, mRNA 16, plasmid DNA 17, or introduction of the recombinant proteins into somatic cells 16. Also, small molecules 18 can substitute some reprogramming genes. Interestingly, microRNAs (miR) 19,20 can produce iPSCs on their own, without the pluripotency reprogramming genes. It is especially relevant the ability of miR-302 to contribute to iPSCs generation, which is in line with the well-established role of miR-302 as a key regulator of pluripotency in hESCs 21–25.

However, the generation of iPSCs is still inefficient, yielding a low proportion of reprogrammed cells 18. Many small molecules and epigenetic remodeling drugs have been reported to increase iPSC efficiency 26. However, how these ectopic factors can make a somatic cell to travel back in development and become pluripotent through epigenetic reprogramming remains elusive. Cellular reprogramming is assumed to be a stressful cellular process. In fact, during the reprogramming process several genomic insults including coding mutations, insertions/deletions (indels), and chromosomal rearrangements occur. This genomic instability may activate the DNA damage response (DDR) which may in turn contribute to a selection of specific clones during reprogramming to pluripotency 27–29. Also, whether or not induced pluripotency is a purely stochastic process remains controversial 30. There are important regulators of this process, such as p53 31–33 and Mbd3. Indeed, inhibition of Mbd3 allowed a reprogramming efficiency of almost 100% 34. Recently, differences in cell cycle length allowed the identification of rapid-cycling cells as responsible for the bulk of reprogrammed cells 35. Together, although many technical and biological questions about the cellular, molecular, and epigenetic mechanisms underlying reprogramming remain elusive, making induced reprogramming an obscure and low efficient process, human iPSCs (hiPSCs) have revolutionized biomedical research opening up unprecedented avenues in developmental biology, drug screening, and disease modeling. Because access to healthy or diseased human neural tissue is a challenge and limits our understanding on neurogenesis and neurodegeneration, diseases affecting the central nervous system might particularly benefit from iPSC biology.

## Neural Differentiation of PSCs

**Figure 1:**
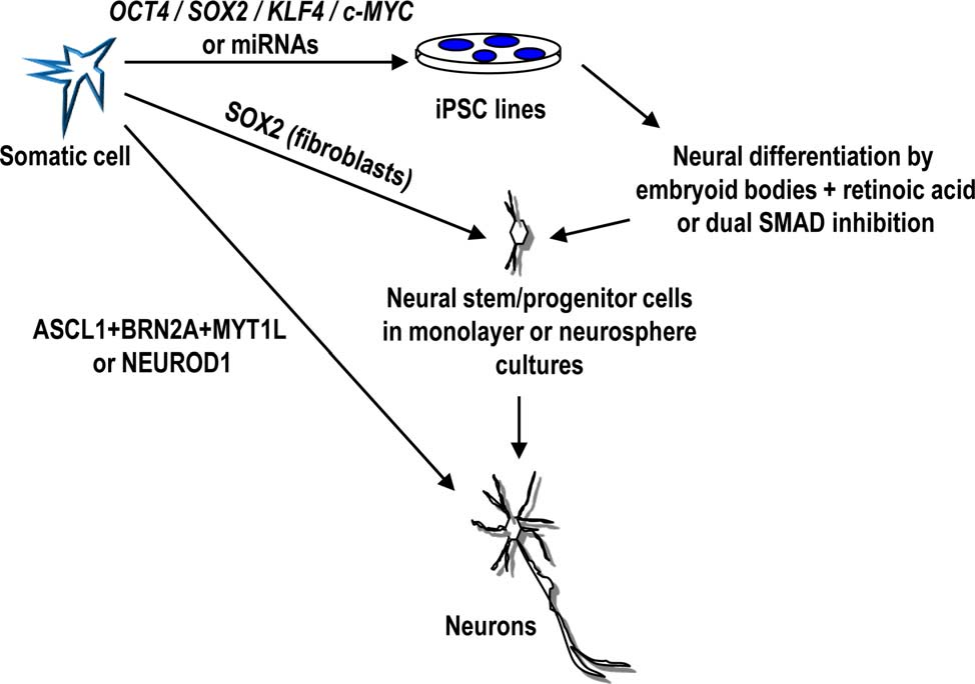
Scheme showing the different methods to produce neurons from a somatic non-neuronal cell. Neural differentiation from human iPSCs requires neural inducers, such as formation of embryoid bodies combined with stimulation with retinoic acid or inhibition of transforming growth factor-β and bone morphogenic proteins (dual SMAD inhibition), followed by expansion of neural stem/progenitor cells in monolayer or neurosphere cultures. Alternatively, fibroblasts, cord blood cells, or astrocytes can be directly converted to neurons, either in vitro or in vivo. Abbreviations: miRNAs, microRNAs; iPSC, induced pluripotent stem cell.

Several protocols for neuronal differentiation of human PSCs, including iPSCs, have been reported. Neural commitment of PSCs can be achieved through formation of embryoid bodies and treatment with retinoic acid, a neuroectoderm inducer, or by pharmacological inhibition of transforming growth factor-β and bone morphogenic protein pathways (dual SMAD inhibition). The resulting neural stem/progenitor cells can be expanded either attached to a substrate or as floating neurospheres to be further exposed to growth factors inducing specific neuronal subtypes (i.e., spinal motor, cerebellar, dopaminergic, or cortical interneurons) 36–40 (Fig.1). For instance, motor neuron differentiation of hESCs and hiPSCs has been refined to a point that spinal lateral column phenotypes can be obtained 36,41. Similarly, for dopaminergic neurons, midbrain phenotypes can be successfully produced in high yields from hESCs and hiPSCs 38,39. These human PSC (hPSC)-derived neurons were transplanted in animal models of neurological disease where survived and contributed to the partial recovery of several neurological parameters, including behavior. These results should encourage us to further explore hPSC-derived neurons in preclinical settings 42. Beyond potential cell therapy applications, hPSC-derived neurons open up new avenues in drug screening and disease modeling applications.

Induced PSCs have been generated from biopsies of patients harboring mutations associated to amyotrophic lateral sclerosis 41, spinal muscular atrophy 43,44, Parkinson disease 45,46, Alzheimer disease 47, and Rett syndrome 48. Some of these patient/disease-specific iPSCs give rise, under proper inductive conditions, to the affected neuronal subtype, opening up new avenues to explore: (a) the potential of these neurons to graft the patient in an autologous setting, provided the procedure is safe and effective, (b) the in vitro neuronal differentiation of mutated iPSC as a disease model to unravel the developmental, cellular, and molecular mechanisms underlying the disease onset and progression, and (c) large-scale drug screening aimed at restoring impaired functions or preventing neuronal degeneration. In fact, long-term culture of dopamine neurons differentiated from mutant Parkinson hiPSCs showed increased apoptosis and decreased number and shorter neurites 45. In another study, Parkinson patient-specific iPSC were generated and differentiated into neural derivatives that were challenged with mitochondrial stressors, revealing an increased susceptibility as compared to neural cells from control iPSCs. Furthermore, supplementation of the culture medium with the antioxidant coenzyme Q10 or rapamycin caused partial protection against neural degeneration 49. These studies underscore the potential of iPSCs and iPSC-neural derivatives to gain insights into the underlying mechanisms of neurodegeneration and to explore them as a biological platform to screen potential therapeutic molecules, eventually contributing to the development of novel pharmacological approaches.

Collections of patient/disease-specific hiPSCs, including neurological conditions 50, will be of utmost value to advance our understanding of the disease. Furthermore, an initiative for public banking of diseased fibroblasts from patients with mutations related to neurodegenerative diseases is already ongoing 51. It will be important to consider both sporadic and familial forms of neurological diseases to allow comparisons among patients and mutations in order to identify common and distinct mechanisms underlying the disease pathogenesis as well as wide-acting drugs. However, the generation of functional neurons from hPSCs, namely cells that express markers of neuronal differentiation (β-III tubulin, microtubule associated protein-2, or NeuN), fire action potentials, synthesize and release neurotransmitters and form synapses with neighboring neurons, often requires extended periods of culture and the available protocols are complex and suffer from high variability. Recently, lineage priming to neurons from hESCs and iPSCs was induced by forced expression of a single transcription factor (either NEUROGENIN-2 or NEUROD1) which resulted in the emergence of mature neurons within 2 weeks, speeding up significantly the time required to obtain mature human neurons in vitro 52. Besides differentiation efficiency, a homogeneous population of lineage-specific cells (i.e., neuronal populations) from hPSCs must be eventually obtained to prevent the presence in the culture of residual undifferentiated cells which may exert undesired/misleading effects both in vitro and in vivo.

## Direct Conversion of Somatic Cells Into Neurons

After the seminal reprogramming work of Yamanaka and coworkers, other investigators started testing the hypothesis that expression of transcriptional regulators might act as neural cell determinants to non-neural differentiated somatic cells. Indeed, ectopic expression of ASCL1, BRN2A, and MYT1L turned fibroblasts directly (no pluripotent stage involved) into induced neurons (iNs) 53,54 (Fig.1). Expression of additional neural lineage-specific transcriptional regulators further promoted the conversion of human fibroblasts into dopamine 55,56 and motor neurons 57. Interestingly, although this direct conversion into iNs rarely requires the neural progenitor state, human fibroblasts can also be induced by the sole expression of SOX2 to become multipotent neural stem cells, able to differentiate into neurons, astrocytes, and oligodendroglia 58. Direct conversion of fibroblasts into iNs usually requires a skin biopsy that is expanded in culture to generate enough starting material. Therefore, the use of a more accessible source in medical diagnostic procedures, such as blood, would be highly advantageous. In fact, cord blood stem cells have been recently reprogrammed into iNs by forced expression of SOX2 and c-MYC 59. This work showed for the first time that cells from mesodermal origin can be switched to an ectodermal fate with only two transcription factors. The procedure for generating iNs relies on the use of integrative vectors, and similar to the production of hiPSCs, is rather complex and inefficient. Recently, some progress has been made in direct reprogramming of non-human primate fibroblasts into region-specific neural progenitors through transient expression of the four Yamanaka factors using nonintegrative SeV in combination with specific neural culture conditions 60. These results represent an important technical progress for the generation of iNs, although the presence of a pluripotent intermediate cellular stage cannot be ruled out in these experiments, and the process continues to be quite inefficient. Because SeV vectors seem to be one of the most efficient nonintegrative strategies for reprogramming blood cells to iPSCs 61,62 it will be of interest to determine if delivery of SOX2 and c-MYC by means of SeV transduction might enhance the direct reprogramming efficiency of blood cells into iNs. Blood-derived iNs could provide a unique tool for drug screening and will facilitate the development of a cellular platform for the generation of patient-specific neuronal cells for future biomedical applications.

Direct conversion of non-neuronal cells into neurons is also possible in vivo. Human fibroblasts or astrocytes engineered to express doxycycline-inducible *ASCL1*, *BRN2A*, and *MYT1L* are converted into neurons within the rodent brain after doxycycline addition. Interestingly, endogenous brain astrocytes were also converted into iNs highlighting a specie-independent direct conversion 63. In a similar approach, NeuroD1 transduction of reactive astrocytes and oligodendrocytes present after acute brain damage or as a consequence of a chronic damage in a transgenic Alzheimer disease mouse model also rendered iNs in vivo. Notably, astrocyte-derived iNS mainly produced glutamatergic neurons whereas oligodendrogyte-derived iNs generated both glutamatergic and gabaergic neurons 64. To what extent neuronal direct conversion is possible in other diseased somatic non-neural cells remains an open question. While these results are promising, the field has yet to clearly address how much these iNs resemble to neurons/neural progenitors and whether iNs retain epigenetic memory; that is, they retain gene expression and epigenetic profiles similar to the donor cell type (fibroblasts, hematopoietic cells and astrocytes) that was originally reprogrammed. This epigenetic memory is crucial since it may influence subsequent differentiation potential. Taken together, integration of technical and biological expertise gained from hiPSCs and hiNs will boost our ability to move the field forward facilitating the implementation of disease modeling and drug screening applications. Eventually, the realization of the full potential of iPSCs/iNs relies on the ability to improve the reprogramming/direct conversion and combine efficient differentiation protocols with the precise modification of specific genome sequences.

## Targeted Genome Editing in Human iPSCs

**Figure 2:**
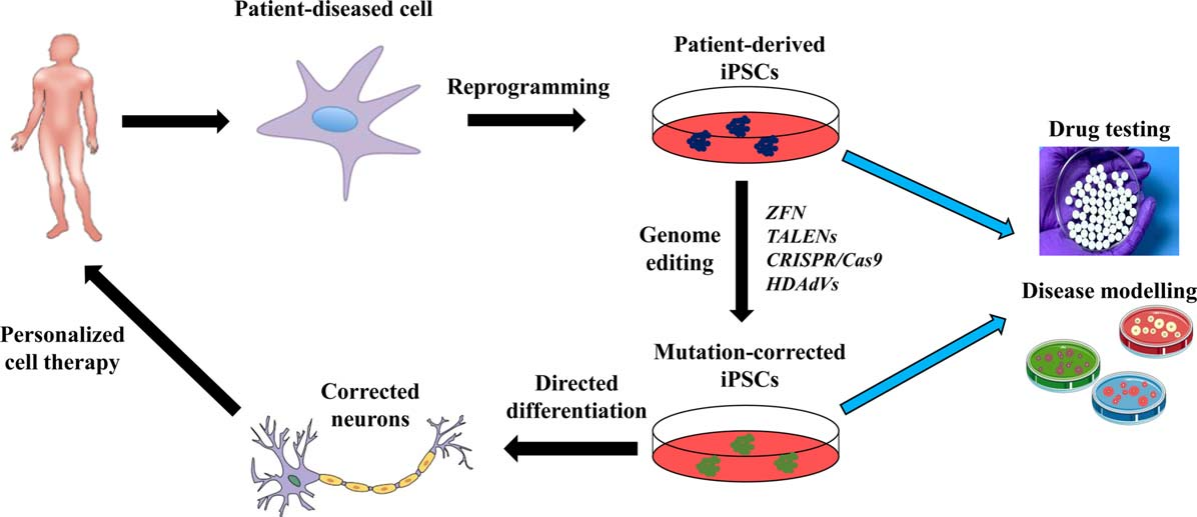
Cartoon depicting the strategy for combining genome editing and patient-specific human iPSCs (hiPSCs) for in vitro generation of diseased and corrected lineage-specific derivatives for disease modeling and drug screening. Studies on disease modeling and drug screening should be undertaken in parallel on both diseased and genetically corrected cell lines. Due to the high variability existing among hiPSC lines derived from distinct starting cells, using different methods, and from different genetic backgrounds, the use of an isogenic mutation-corrected iPSC line is essential as a control. Abbreviations: CRISPR/CAS9, clustered regularly interspaced short palindromic repeat/CAS9 RNA-guided nucleases; HDAdV, helper-dependent adenoviral vectors; iPSCs, induced pluripotent stem cells; TALENs, transcription activator-like effector nucleases; ZFN, zinc finger nucleases.

hiPSCs are widely being used in developmental biology and disease modeling. However, they are envisioned to become a unique tool for disease-specific drug screening, and possibly, a patient-specific cell replacement approach 65 (Fig.2). However, the realization of the full potential of hiPSCs relies on the ability to precisely modify/correct specific genome sequences, with a prospect of personalized cell therapy. Importantly, genome editing in hPSCs has evolved from being a daunting task to a widely spread procedure in worldwide laboratories. The reasons for this are twofold: (a) human PSCs are especially amenable for genome editing since they can undergo extensive culture manipulations, such as drug selection and clonal expansion, while still maintaining their pluripotency and genome stability 66, and (b) genome editing technology has progressed extremely rapid over the last few years including zinc finger nucleases (ZFNs), transcription activator-like effector nucleases (TALENs), clustered regularly interspaced short palindromic repeat/CAS9 RNA-guided nucleases (CRISPR/CAS9) (Table 1), and helper-dependent adenoviral vectors (HDAdVs). An important aspect is that cells that have undergone genome editing should contain only the intended change in an otherwise isogenic background, thus providing the most stringent test of gene function. However, this may not be the case due to off-target effects of ZFNs, TALENs, and CRISPR/CAS9. However, these genome-editing tools are under continuous improvement 67,68. The biological mechanisms underlying these genome-editing tools in human PSCs cannot be covered in this review due to space constrains, but they have been extensively and elegantly reviewed elsewhere 66. Table 1 summarizes the differential biological and technical features of cutting-edge main genome-editing approaches. Although only a minority of the neurodegenerative diseases is caused by a specific identified mutation, there are examples showing the potential of genome editing in iPSC for neurological diseases.

**Table 1:** Main biological-technical features of genome-editing technologies

Feature	ZFNs	TALENs	CRISPR/CAS
Type of recognition	Protein–DNA	Protein–DNA	RNA–DNA
Mechanism of action	Induces DSB	Induces DSB	Induces DSB and SSB (nickase)
Design/construction	Intermediate/easy	Simple/easy	Very simple/very easy
Success rate	Low	Moderate	High
Methylation sensitive	Not sensitive	Sensitive	Not sensitive
Multiplexing/high-throughput	No	Feasible (but technically challenging)	Yes
Off-target effects	Common	Few	Common (few with nickase)
Toxicity	Variable	Low	Low
Cost	High	Moderate	Very low

Abbreviations: CRISPR/CAS, clustered regularly interspaced short palindromic repeat/CAS9 RNA-guided nucleases; DSB, double strand breaks; SSB, single strand breaks; TALENs, transcription activator-like effector nucleases; ZFNs, zinc finger nucleases.

Huntington disease is caused by expansion of a poly-glutamine motif in HUNTINGTIN. Through homologous recombination in iPSCs, the number of glutamines in this protein was reduced, resulting in decreased apoptosis and improvement in oxygen consumption rate in corrected neural progenitors 69. Spinal muscular atrophy is a genetic autosomal recessive disease caused by mutations of the *SMN1* gene which results in a nonfunctional protein causing motor neuron degeneration. As a compensatory mechanism, these patients rely on the expression of *SMN2*, which is highly homologous to *SMN1* but has a change in a nucleotide that alters its splicing, with a consequent reduction in the amount of SMN produced. Using single-stranded DNA sequences, editing of the *SMN2* was performed, resulting in a SMN1-like protein that includes the exon 7. This change was shown to be stable and correlated with increased motor neuron survival in repaired cells 43. Recently, ZFN-mediated genome editing and iPSC technology were combined to generate sets of isogenic diseased and control hiPSCs that differ exclusively at either of two susceptibility variants for Parkinson's disease by modifying the underlying point mutations in the α-synuclein gene 46. In addition, a point mutation related to familial Parkinson has been corrected using HDAdVs. Notably, these diseased hiPSCs produced neural stem cells that showed alterations in the nuclear architecture, a finding that was not previously reported as related to this pathology. Genetic correction of hiPSCs resulted in normalization of the nuclear alterations, decreased susceptibility to apoptosis, as well as increased neuronal production 70. The robust capability to genetically correct Parkinson disease-causing point mutations in patient-derived hiPSCs represents a noteworthy progress in basic neurosciences, and a significant advance toward hiPSC-based cell replacement therapies and drug testing. Whether or not the corrected neurons will survive after transplantation in the altered central nervous system (environment) represents a next level of complexity that still needs to be addressed.

## Conclusion

Over the last decade we have witnessed significant progress in both our understanding of cell fate determinants and also in the possibilities to use reprogramming in the preclinical setting. The generation of human neurons by these methodologies will definitively allow the analysis of the mechanisms causing familial and sporadic cases of neurological conditions. Patients therefore can benefit from in vitro differentiated neurons and specific drugs identified by high-throughput screening. If genome editing turns out to be safe and effective, we envision a future prospective production of clinically relevant neuronal types within the brain of affected people. Whether or not the synaptic communication will be re-established properly in the diseased brain is a different question, but repopulation of the affected neuronal phenotype, either by transplantation of differentiated neurons or by direct conversion of resident glial cells is a very encouraging step forward.
